# Facile fabrication and biological investigations of metal oxides intercalated in kaolinite clay-based dressing material to improve wound healing ability in nursing care after post-operative period

**DOI:** 10.1016/j.heliyon.2024.e25289

**Published:** 2024-01-26

**Authors:** Xia Cheng, Jingjing Yao, Wenhao Fan, Li Zhen

**Affiliations:** a Department of General Surgery, Nanfang Hospital, The First School of Clinical Medicine, Southern Medical University, Guangdong Provincial Engineering Technology Research Center of Minimally Invasive Surgery, Guangzhou, Guangdong Province, China; b Guangdong Provincial Key Laboratory of Precision Medicine for Gastrointestinal Tumor, Guangzhou, Guangdong Province, China; c Department of Hematology, Nanfang Hospital, The First School of Clinical Medicine, Southern Medical University, Guangzhou, Guangdong Province, China

**Keywords:** Nanocomposite, Cell viability, Wound healing, Regenerative medicine, Tissue engineering

## Abstract

The present investigation aims to design and development of hybrid zinc oxide (ZnO) and manganese dioxide (MnO_2_) nanoparticles (NPs) doped-biopolymer matrix-based cost-effective technique for the synthesis of biocompatible Kaolinite/Chitosan (Ka/CS) nanocomposites (NCs) could be used as agents for wound healing due to their efficiency and low toxicity. The crystallite size, phase purity and surface morphology of the synthesised NCs were investigated systemic analytical methods. The results revealed that the metal oxide nanocomposites presented that in rod-crystalline in shape and additionally exhibits that 20–30 nm in size. *In vitro* antibacterial analyses demonstrates that NCs have significantly improved bactericidal inhibition efficiency when compared to the bare hybrid NPs and polymeric components. The *in vitro* biocompatibility observation demonstrates that prepared hybrid-NPs encapsulated NCs have enhanced cell survival rate (>90 %), which was established by MTT assay and Live/Dead fluorescence assay methods at different incubation time. The DPPH assay was used to investigate the synergistic effects of prepared dressing materials increased antioxidant activity. Preliminary research indicates that these nanocomposites, ZnO/MnO_2_ incorporated and decorated with Ka/CS NCs, could be a significant promoter and potential candidate for use as a robust wound healing agent in post-operative nursing care.

## Graphical abstract

**Figure:**
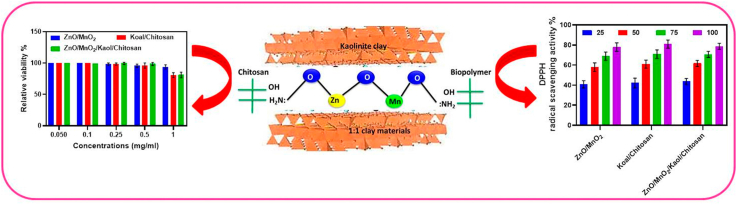

## 1 Introduction

Wound infection and tissue engineering *in vitro* are a common and puzzling issue in wound healing structural and functional properties of tissues. With the spread of antibiotic-resistant bacteria, any category of wound on the skin would be susceptible to the acclamation of infection area [1,2]. Using antibiotics to prevent and treat wound infections in inefficient, so it imposes added expenses on the patient and society. As a result, alternative materials are essential in the struggle against infections [3,4]. In pharmacological applications since ancient times, clay minerals are a well-known skin heals wounds with minimal or lack of interference [5,6]. Because of their nontoxic, swelling properties, environmental friendliness, and charged surfaces which can be easily modified through an intercalation process; clay minerals are captivating attention inorganic hosts materials for functional inorganic-organic hybrids. Kaolinite is the most common clay mineral, it has layers similar to those of gibbsite with aluminium and silica atoms (1:1) uncharged di-octahedral and tetrahedral coordinated with four oxygen atoms and each layer consists of single Si and Al oxides [7]. Clays-modified have been successfully and developed used as antibacterial, antioxidant and all bioactive *in vitro* applications [[8], [9], [10], [11]]. To enhanced stability of Kaol clay, the functional groups amine (-NH_2_) could be utilised, such as chitosan (CS) [12,13].

The development of novel materials with therapeutic uses is a significant concern within the field of biomedical engineering. Nanotechnology has facilitated the development of nanoparticles that possess exceptional physicochemical characteristics, such as a higher aspect ratio and a significantly larger surface area to volume ratio when compared to their bulk counterparts [14,15]. Nanomaterials can be categorised into two distinct classes based on their origins: organic and inorganic forms. Inorganic nanoparticles include a variety of metal and metal oxide nanoparticles, including Ag, Cu, CuO, ZnO, TiO_2_, Fe_3_O_4_, and MgO NPs [5,16,17]. In recent times, there has been an increased focus on the medicinal and industrial significance of these NPs. Numerous researches have demonstrated the presence of antibacterial, anticancer, antidiabetic, and wound healing effects in ZnO nanoparticles. The observed characteristics of ZnO nanoparticles can be attributed to their photocatalytic capabilities and the generation of reactive oxygen species (ROS) [18,19]. The development of bioactive composite hydrogels doped with zinc has been undertaken for the purpose of wound closure, with the aim of establishing a conducive milieu that promotes the proliferation of cells involved in the wound healing process. The antibacterial effects of composite hydrogels composed of succinyl chitosan and Zn^2+^ were observed [[20], [21], [22]]. Nevertheless, the utilisation of composite hydrogels necessitates the structural alteration of chitosan in order to enhance its solubility. Furthermore, the inadequate mechanical characteristics of these hydrogels impose restrictions on their practicality in the context of wound dressings [23,24].

Furthermore, the presence of harmful reactive oxygen species (ROS) creates an oxidative wound microenvironment that impedes the efficient healing of wounds. Therefore, there is a strong demand for a single platform that can effectively eradicate ROS while simultaneously providing a constant supply of oxygen [25]. The catalytic properties of manganese dioxide (MnO_2_) have been extensively studied, revealing their effectiveness in decomposing endogenous hydrogen peroxide (H_2_O_2_) into oxygen (O_2_). This catalytic activity has been found to alleviate oxidative stress and hypoxia, making MnO_2_ nanosheets a highly promising nanomaterial in this regard [26,27]. In contrast to other nanomaterials such as nanoceria (CeO_2_), gold nanoclusters, and C_3_N_4_ nanofusiform, MnO_2_ nanoparticles exhibit biodegradability and biocompatibility [[28], [29], [30]]. However, the use of MnO_2_ nanoparticles in anti-cancer therapy or wound healing is hindered by several factors, including their uneven distribution, early depletion before reaching the intended locations, and inadequate retention properties [31]. Hydrogels have demonstrated significant potential in facilitating the extended retention, sustainable release, and profound penetration of encapsulated compounds. Hence, the integration of MnO_2_ nanosheets into a suitable injectable hydrogel holds significant potential as a valuable strategy for modifying the detrimental microenvironment. This approach effectively addresses the concurrent requirements of targeted multimodal melanoma eradication and skin tissue regeneration [[32], [33], [34]].

Chitosan (CS) is widely recognised as a polysaccharide that possesses biocompatible, hemostatic, and non-toxic properties. Consequently, it is highly regarded for its potential in drug delivery systems, particularly in the development of wound dressings and tissue engineering scaffolds [[35], [36], [37]]. Chitosan, a naturally occurring co-polymer composed of deacetylated derivative of chitin units (β-(1 → 4)-linked d-glucosamine) and units that remain un-deacetylated (*N*-acetyl-d-glucosamine), has garnered significant interest among researchers. The foundation of this statement pertains to the notable characteristics of chitosan, which include its biocompatibility, biodegradability, bio-functionality, antibacterial activities, and non-toxic nature. The wound dressing composed of chitosan (CS) demonstrates many characteristics that contribute to the process of wound healing. These qualities include the stimulation of fibroblast proliferation, the production of collagen, the enhancement of inflammatory cell activities, and the engagement of integrins. Hence, it is imperative to undertake chemical alterations to the structure of CS in order to enhance its physicochemical qualities for further utilisation [12,38,39]. The CS polymer is a modified form of CS that exhibits the ability to interact with cells, leading to favourable outcomes in terms of cell development and wound healing. Chitosan is employed in the field of cosmetics due to its stabilising agent features, moisture absorption and retention capabilities, and antibacterial characteristics [13,40,41]. Therefore, it is reasonable to anticipate that the amalgamation of CS and ZnO could yield a synergistic antibacterial outcome while maintaining non-toxicity towards regular cells, thereby potentially augmenting the process of wound healing.

To the best of our knowledge, there are no previous reports on a hemostatic, biocompatible, and antibacterial MnO_2_–ZnO nanocomposite created using Kaol-Chitosan. The structural characteristics of the CS- ZnO nanocomposite were investigated by various characterization techniques. Furthermore, the biological properties of the CS-ZnO nanocomposite. In this study, the ZnO–MnO_2_, Kaol/Chit and ZnO–MnO_2_/Kalo/Chit nanocomposites were synthesised and characterized by XRD, FT-IR, TEM, and SAED technique and bioactive activity of nanocomposites were studied. The wound models were subjected to histological analysis, wound healing measurement day by day, and various biomedical applications for post-operative nursing care.

## 2 Experimental details

### 2.1 Materials

Merck Chemicals Ltd provided kalonite clay – free from toxic element, Zinc nitrate (Zn (NO_3_)_2_, Manganese nitrate hexahydrate (Mn (NO_3_)_2_.6H_2_O), Sodium hydroxide (NaOH), and Sigma-Aldrich supplied Chitosan (medium molecular weight) (31,00,000–85 % decaetylated) China P.R., which were utilised without additional purification or treatment. Throughout the experiment, double deionized (DI) water was used. Kaolinite chemical composition consisted of SiO_2_ - 54.0, Al_2_O_3_ – 31.7, K_2_O - 6.05, TiO_2_ – 1.41, Fe_2_O_3_ - 4.89, MnO_2_ - 0.11, ZrO_2_ - 0.10 and LOI -1.74.

### 2.2 Synthesis of ZnO/MnO_2_/Kaolinite/Chitosan NCs

Step 1 To synthesis of Kaol/Chit NCs, 1 g of Kaol and 2g of Chit solution (acetic acid 1 %) solution were mixed with DI water. The resulted solution was stirred for 24 h at room temperature. Kaol/Chit nanocomposite was obtained.

Step 2 In a typical reaction procedure, 50 ml of 0.2 M aqueous Zn(NO_3_)_2_ and Mn(NO_3_)_2._6H_2_O solutions were mixed and stirred continuously by added 0.4 M of NaOH (molar ratio - 1:4) into the solution for 4 h. A precipitate consisting of hybrid nanoparticles of ZnO and MnO_2_ was collected. Following the removal of byproducts via centrifugation and washing, the precipitate was dried at 300 °C. The hybrid nanoparticles were collected for further use.

Step 3 The nanoparticles was dispersed in DI water and added into solution A to prepare ZnO/MnO_2_/Ka/CS NCs. Subsequently, the pH of the mixture was adjusted to 7.4–7.5 and the solution A and nanoparticles solution were stirred together vigorously at half an hour. The solution mixture was placed in a 100 mL Teflon-lined stainless-steel autoclave 120 °C for 10 h. To isolate the resulted product from solution mixture 'C' throughout samples were centrifuged after the reaction hours have been completed. The obtained precipitate was washed with distilled water and ethyl alcohol for several times in order to remove impurities ions in the obtained products ZnO/MnO_2_/Kaolinite/Chitosan NCs. It was ultimately dried at 100 °C for 3 h.

### 2.3 Characterization

The X-ray diffraction (XRD) observations were performed using nickel-filtered Cu k radiation (– 1.54178) on a Dmax-3 diffractometer. Employing an accelerating voltage of 10 kV, the surface microstructure of the composite materials were investigated using a JEOL JSM 639-SEM equipment. Transmission electron microscopy with chosen area electron diffraction was used to examine the morphology and nanostructure of the products (TEM with SAED patterns, Tecnai F20). Using the KBr pellets, the Fourier transforms infrared spectroscopy (FT-IR) spectra of samples were recorded on the Shimadzu (model-400) from 4000 to 400 cm^−1^.

### 2.4 Antibacterial assessment

To test the antibacterial performance of ZnO–MnO_2_, Kaol/Chit, ZnO–MnO_2_/Kaol/Chit NCs containing varying amounts of samples and sterilised in 75 % ethanol for 30 min before being washed twice with phosphate buffer saline (PBS). The bacterial pathogens such as Staphylococcus aureus (S. aureus) and Escherichia coli (E. coli) strains were mixed to around 2 × 10^8^ CFU/mL in an atmosphere that was sterile. A 2 mL as-prepared E. coli suspension was used to immerse six films for four days at 4 °C. After diluting the bacteria suspension 6 × 10^4^ times, a 15 μL portion was used to cover LB plates and was left to grow at 37 °C for 24 h subsequently, a digital colony counter and a UV–Vis spectrophotometer were employed to determine the number of bacterial colonies forming units.

#### 2.4.1 Analysis of growth curve

The growth curve experiment is a useful tool for determining NCs materials’ antibacterial activity. The antibacterial activity of the synthesised ZnO–MnO_2_, Kaol/Chit, ZnO–MnO_2_/Kaol/Chit NCs was investigated using a standard micro dilution method, which resulted in bacterial growth inhibition by agar diffusion method. The bacterial cultures were incubated in an orbital shaker at 37 °C after being introduced to a flask containing 10 mL of LB Lennox medium. The concentrations of the strains ranged from 10 to 20 μg/mL. Using a UV-Cis spectrophotometer, the optical density (OD) of the test bacterial strains in broth was measured at 595 nm at regular intervals ranging from 0 to 24 h. The experiments were duplicated and then conducted twice more. A growth curve involving OD and time has indeed been graph generated.

#### 2.4.2 *In vitro* biofilm analysis

To confirm the samples' antibacterial biofilm effects, we seeded each bacterial cell type onto a poly-l-lysine-coated cover glass and incubated it for 1 h. Prior to incubating with samples for 24 h, the cover glass was rinsed three times with a 0.9 % saline solution to eliminate any detached cells. Furthermore, the acridine orange kit was used to stain both living bacterial cells, according to instructions by the manufacturer. A confocal fluorescent microscope (Olympus, Japan) was utilised to analyse. Every fluorescence image was examined using ImageJ software.

### 2.5 *In vitro* antioxidant assay (DPPH assay)

The free-radical scavenging activity of antioxidants is commonly measured using DPPH assay. Reduced absorbance caused by antioxidants was used to investigate the DPPH radicals reduced capacity. The reaction system contained 0.1 mL ZnO–MnO_2_, Kaol/Chit, ZnO–MnO_2_/Kaol/Chit nanocomposites diluted to various concentration (25, 50, 75, and 100 μg/mL), as well as 2.9 mL of 0.025 g/L DPPH in DMSO. The inhibition percentage (%) was measured by using the following equation (1);

(1) $$Inhibition\;\left(\%\right)=\left[\frac{\left(Ab-As\right)}{Ab}\right]\times 100$$

In this instance, Ab and As indicate the blank and sample absorbance, respectively.

### 2.6 *In vitro* cell compatibility assay

NIH-3T3 cells (10^4^ cells/well) were tested for cell viability using ZnO–MnO_2_, Kaol/Chit and Kaol/Chit/ZnO–MnO_2_ nanocomposites. The toxicity of the produced ZnO–MnO_2_, Kaol/Chit and Kaol/Chit/ZnO–MnO_2_ nanocomposites were evaluated *in vitro* using NIH 3T3 cells within a week of 1, 3, 5, 7 and 12 h. Each group's precursor mixture was placed in a 96-well plate and solubilized for 3 h at 37 °C in a sterile environment. The scaffolds were seeded with 1–2 x 10^5^ NIH-3T3 cells per well and culture in DMEM supplemented with 10 % FBS medium and 1 % anti-biotics. Cell Potential was measured using the MTT assay at pre-specified time points after the cells had indeed been cultured for 24 h in a humidified 5 % CO_2_ incubator. Each well was treated overnight at 4 h intervals with MTT (50 L of 5 mg/mL in PBS) after the medium had been removed for 24 h. Thermo Scientific's Scanning Multimode Reader was used to measure the absorption spectra at 570 nm after 150 L of DMSO was added to formazan crystals as an emulsifying buffer. As a 2D control, cells sown in a well without any sample were used. The cell survival percentage was calculated by the following equation (2);

(2) $$Percentage\;of\;Cell\;viability\;\left(\%\right)=\frac{{OD}_{Treated}\;}{{OD}_{Control}}\times 100\;\%$$

## 3 Results and discussion

### 3.1 Possible mechanism of M − O NCs intercalated into kaolinite with biopolymer

In a typical green route, kaolinite clay with chitosan biopolymer mixture well without foaming and continuously stirred for 24 h at room temperature and separately stirred ZnO–MnO_2_ NCs synthesised from the stock solutions of zinc nitrate (Zn(NO_3_)_2_, and manganese nitrate hexahydrate (Mn(NO_3_)_2_.6H_2_O) for half an hour at ambient temperature. Constantly two composite solutions were mixture well and then relocated the 100 mL tiffon-lined stainless autoclave 120 °C for 10 h for achievement of the functionalized or incorporated ZnO–MnO_2_ NCs into Kaol/Chit NCs. The incubated reaction mixture was then dried hot air oven at 100 °C for 3 h [5]. Completely dry intercalated into Kaol/Chit NCs. Above resulted mechanism, {(Chit---(HN)-----[O-----Mn-----*O*-----Zn-----O]----(:NH_2_)--Chit----Kaol)} metal---oxy---metal bond is electrostatic interaction with surface oxygen in Kaolinite and lone pair of electron from nitrogen in chitosan. The obtained ZnO/MnO_2_/Kaolinite/Chitosan NCs powder was crushed and placed in Eppendorf's for further utilisation in characterization and biological studies.

### 3.2 XRD

**Fig. 1:**
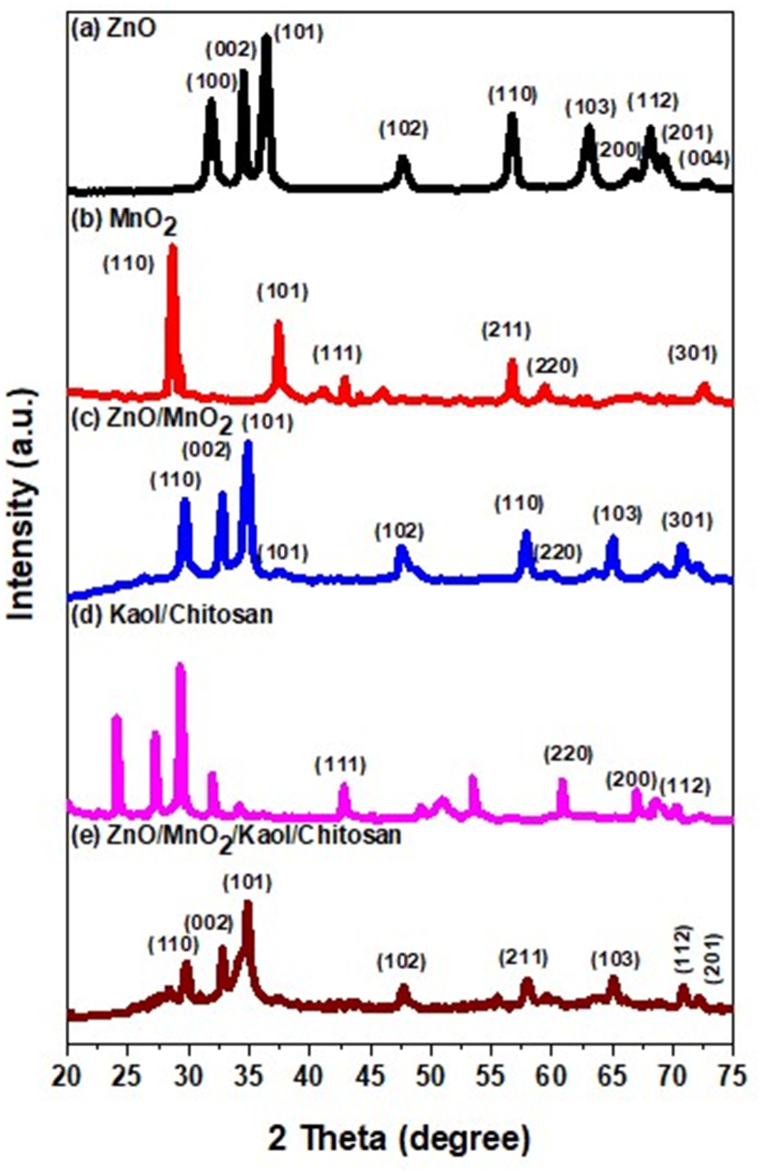
XRD diffraction pattern analyses for ZnO NPs, MnO_2_ NPs, ZnO/MnO_2_, Ka/CS and ZnO/MnO_2_/Ka/CS NCs.

**Fig. 2:**
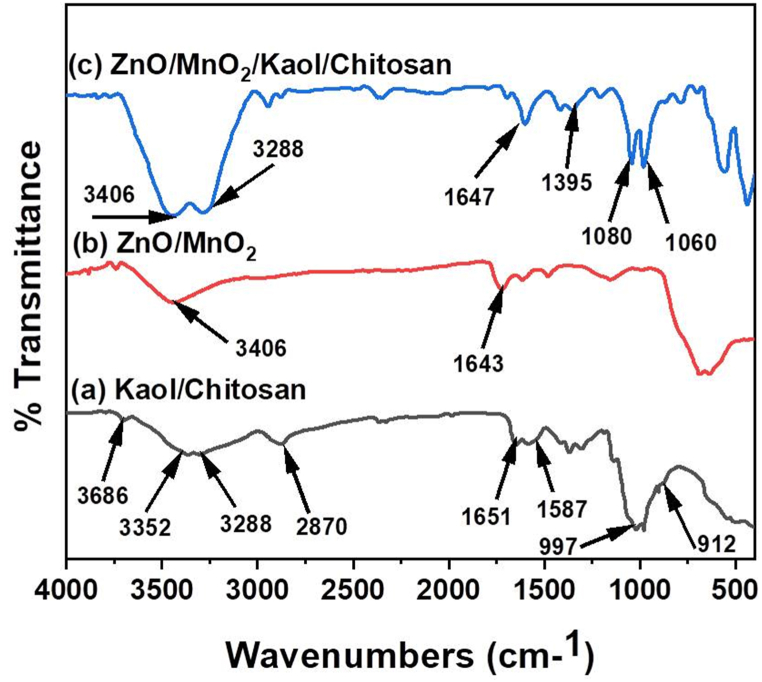
FT-IR analysis of ZnO/MnO_2_, Kaol/Chit and ZnO/MnO_2_/Kaol/Chit NCs.

XRD pattern for the prepared nanocomposite could be seen in Fig. 1. XRD pattern of ZnO/MnO_2_, Kaol/Chit contained diffraction peaks of ZnO/MnO_2_/Kaol/Chit NCs. The XRD peaks and corresponds hkl planes at 2Ѳ = 31.6° (100), 34.8° (002), 36.6° (101), 37.6° (211), 46.1° (102), 56.4° (600), 57.1° (110), 63.2 (103), 68.9 (112) and 71.3 (222) regarding for hexagonal ZnO/tetrahedral α-MnO_2_ NCs - and (JCPDS Card No: 76–0704 and 44–0141 in Fig. 1a) phase, respectively. The diffraction peaks and corresponding to the reflections of hkl planes at 2Ѳ = 12.4° (001), 19.8° (020), 23.8° (021), 24.9° (002), 31.9° (241), 32.6° (022), 33.9° (152), 35.7° (143), 47.7° (113), 56.1° (024), 57.4° (043), 66.6° (242), 71.5° (153) are related to End-Centered Kaol/Orthorhombic Chit NCs and (JCPDS Card No:74–1784 and 33–1894 in Fig. 1b) phase. The prominent diffraction peaks at 2Ѳ = 31.5°, 33.8°, 35.9°, 47.8°, 56.2°, 57.7°, 66.6°, and 71.4°, these peaks are well are agreed in ZnO/MnO_2_/Kaol/Chit NCs in Fig. 1c. Using Debye Scherrer's equation, D = 0.9xλ/(dCosѲ), the average grain size generated in the synthesis NCs was estimated to be (a) 30 nm, (b) 35 nm, and (c) 25 nm for the higher intensity peak. The crystallite size for ZnO/MnO_2_/Kaol/Chit NCs was 21.5 nm is small crystalline size than other nanocomposite due to increase of crystalline structure of nanocomposites which appeared in form of highly sharp peaks shown in Fig. 2. Both NCs exhibited an absence of impurity bands and secondary phases in their diffractograms, indicating that the synthesised material was pure. As a result, ZnO/MnO_2_ NCs incorporated into the kaolinite clay with capping agents of Chitosan (biopolymer). This can be because the reaction of the environment was due to the presences of electrostatic interaction force in between the ZnO/MnO_2_ and Kaol/Chit NCs [36,40].

### 3.3 FTIR

In Fig. 2, we observe the FTIR peaks that are exclusive to ZnO/MnO_2_, Kaol/Chit, and ZnO/MnO_2_/Kaol/Chit NCs. The vibration mode of metal-oxygen (Zn⋯O/O⋯Mn⋯O stretching vibrations) corresponds to the absorption peaks seen between 3406 cm^−1^ and 1643 cm^−1^. Primary amines' C–N bond stretching vibrations and primary alcohols' O–H bond stretching vibrations are responsible for the peaks at 3686 cm^−1^, 3352 cm^−1^, and 3288 cm^−1^, respectively. Peak absorption at 2870 cm^−1^ is due to the polysaccharide's C–H bond stretching vibrations; peaks at 1651 cm^−1^ and 1581 cm^−1^ are chitosan's C–O, C–*O*–C, and O–H stretching vibrations, as seen in Fig. 2b and a, respectively. We preferred 997 cm^−1^ for the chitosan amine group and 912 cm^−1^ for the Al–O and Si–O stretching vibration bonds, respectively. Fig. 2c illustrates the NCs made of ZnO, MnO_2_, Kaol, and Chit as measured by FTIR spectra. Peaks at 3406 cm^−1^ and 3288 cm^−1^ were determined to correspond to the primary amines' C–N bond and the primary alcohols' O–H bond stretching vibration, respectively, in chitosan. O–H, C=O, and C–*O*–C groups were linked to the peaks at 1647 cm^−1^ and 1395 cm-^1^. Intercalated with kaolinite clay, the peaks at 1080 cm^−1^ and 1060 cm^−1^ indicated M − O (metals bond with oxygen). Because of the ZnO/MnO_2_ and Kaol/Chit NCs, the ZnO/MnO_2_/Kaol/Chit NCs spectra showed a combination of absorption peaks.

### 3.4 SEM and TEM with SAED pattern

**Fig. 3:**
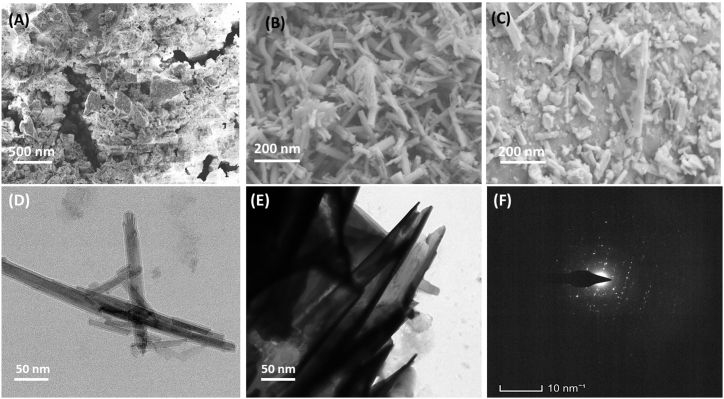
The morphological analysis was observed by SEM and TEM images; SEM morphological observations of (A) Ka/CS, (B) ZnO/MnO_2_ and (C) ZnO/MnO_2_/Ka/CS NCs and (D & E) TEM images ZnO/MnO_2_/Ka/CS NCs with SAED patterns (F).

The surface morphology of ZnO/MnO_2_ NCs was rod-like, and the nano shaped was evenly distributed throughout the Ka/CS (Fig. 3A), ZnO/MnO_2_ (Fig. 3B) and ZnO/MnO_2_/Ka/CS NCs (Fig. 3C). With chitosan acting as a capping agent, a large number of spherical round shaped nano ZnO/MnO_2_ NCs are uniformly intercalated into and onto flattened layers plate like structure of kaolinite clay. Fig. 3 (D & E) shows TEM images of nanocomposites that have been completely intercalated. In addition, as shown in Fig. 3a, the ZnO/MnO_2_ NCs were intercalated into the flattened or plate-shaped kaolinite plates, which were subsequently coated with particles that looked like a clump of small spherical particles with a mesoporous entity. TEM pictures for the morphology of ZnO/MnO_2_/Kaol/Chit NCs showed that the ZnO/MnO_2_ nanoparticles on the kaolinite plates ranged from 20 to 30 nm. Fig. 3F shows the SAED pattern indicated the well-crystallized of ZnO/MnO_2_/Kaol/Chit NCs. Chitosan acts as an antioxidant agent through interaction with increasing membrane permeability, cellular membrane, and killing bacteria and increase viability.

### 3.5 *In vitro* analysis of biodegradation stability

**Fig. 4:**
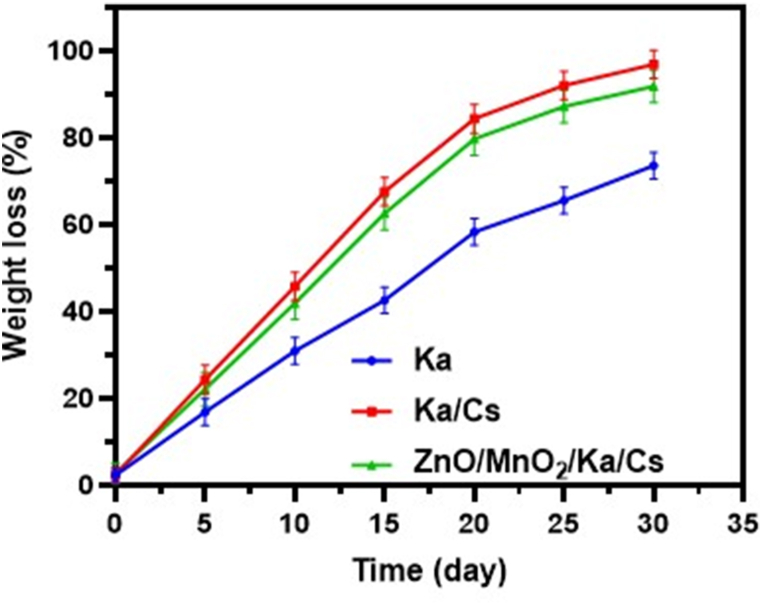
*In vitro* biodegradation stability analysis of prepared Ka, Ka/CS and ZnO/MnO_2_/Ka/CS NCs in different incubation time (days).

To confirm the stability of the NCs, *in vitro* degradability of the samples was assessed by quantifying the reduction in weight under simulated physiological conditions at a temperature of 37 °C. The steady loss of weight from all samples over time is evident, as depicted in Fig. 4. The NCs exhibited a degradation rate of roughly 86 % after a period of 20 days, and achieved near-complete degradation (nearly 98 %) after a duration of 30 days. In contrast, the bare Ka clay compound exhibited a degradation rate of less than 50 % for the corresponding time intervals. Both the nanocomposites, Ka and Ka/CS, exhibited a degradation rate above 70 % after 20 days and 80 % after 30 days when subjected to a PBS environment. Hence, the findings indicate that NCs have significantly accelerated degradation in comparison to the individual components without any further coatings or enhancements.

### 3.6 Antibacterial activity with confocal fluorescence microscopy

The ZnO/MnO_2_/Kaol/Chit NCs that were prepared exhibited a wide range of antibacterial activity against the pathogens that were tested in the present study. The antibacterial effects were confirmed by measuring and plotting the optical densities of ZnO–MnO_2_, Kaol/Chit, and ZnO–MnO_2_/Kaol/Chit NCs as a function of time at regular intervals from 0 to 24 h using the agar diffusion method. The concentrations of the NCs ranged from 10 to 30 μg/mL. Two bacterial strains— such as E. coli and S. aureus—were utilised for evaluating the antibacterial activity for each sample. The antibacterial properties of both samples were evaluated by CFUs of each strain, which is an indicator of the total number of live bacteria.

The antibacterial activity of the ZnO–MnO_2_/Kaol/Chit NCs against *S. aureus* and *E. coli* was impressive. Because of their intercalation complex structure of clay, ZnO–MnO_2_/Kaol/Chit NCs demonstrated effective bacteriostatic and bactericidal properties with the tested organisms. Furthermore, it was demonstrated that the nano O–Zn–Mn–O intercalated into Kaol/Chit NCs had effective antibacterial properties against both bacterial pathogens due to its smaller size. This study hypothesises that the synthesised nanocomposite's small size and outstanding nanostructures provided excellent antibacterial activity [35,42]. The nanocomposites demonstrated the mechanism of the antibacterial property. The release of ions from the nanocomposite, such as Zn^2+^, Mn^4+^, Si^4+^, Al^3+^, O^2−^, and N^3−^, is thought to be responsible for the materials' antibacterial properties [31,43].

**Fig. 5:**
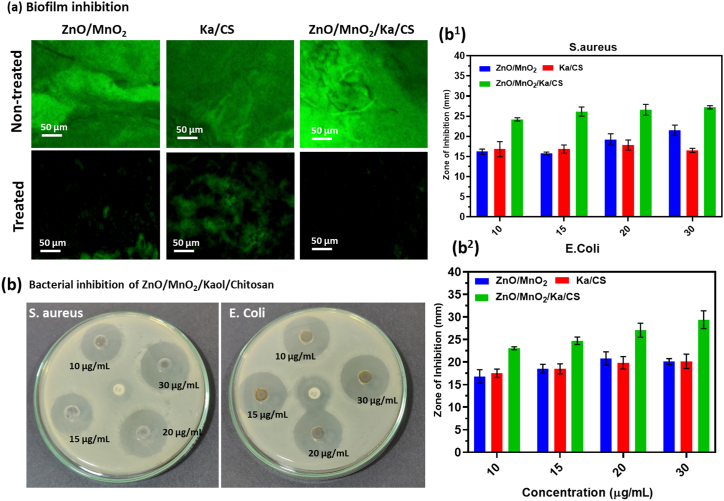
**(a)** The fluorescence microscopic biofilm inhibition images of prepared NCs and NPs against different pathogens (S. aureus and E. coli); (b) Photograph of zone of inhibition plates and (b^1^ and b^2^) bar diagram of ZnO/MnO_2_, Ka/CS and ZnO/MnO_2_/Ka/CS NCs in the different concentration (10, 15, 20, and 30 μg/mL^−1^) against *S. aureus* and *E. coli* pathogens.

**Fig. 6:**
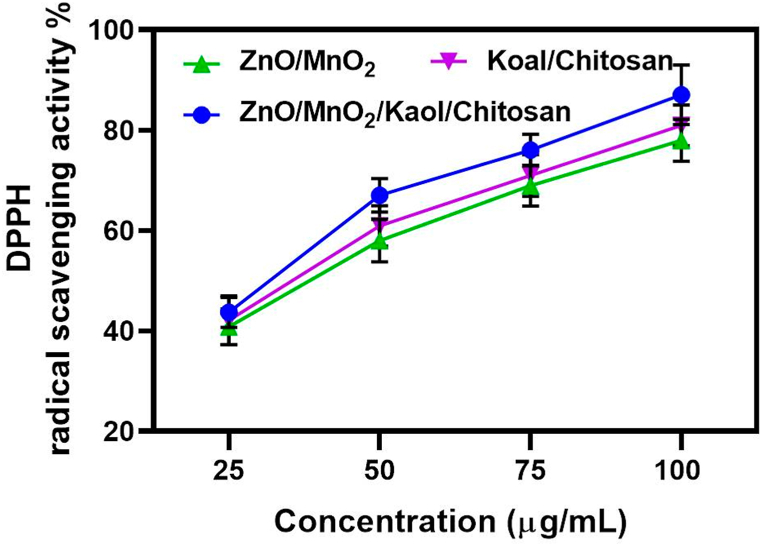
Antioxidant activities of ZnO/MnO_2_, Kaol/CS and ZnO/MnO_2_/Ka/CS NCs against standard DPPH assay.

Furthermore, due to the presence of –CO, –OH, and P groups in lipid bilayers at the cell surface, bacteria cell walls are negatively charged materials in a normal environment. All released cations from intercalated clay materials bind to the above groups, inhibiting normal cellular metabolism and resulting in excellent antibacterial activity in the composites [9,11]. Confocal fluorescence microscopy was used to confirm the selective biofilm inhibition effects, as shown in Fig. 5. When compared to Gram-positive bacteria *S. aureus*, the ZnO/MnO_2_/Kaol/Chit NCs have strong killing effects against Gram-negative bacteria, with complete cell death in the *E. coli* pathogen. According to confocal fluorescence microscopy, the colour green of the living cell is completely distorted. Gram-positive *S. aureus* viability decreased as well, though many cells remained viable in biofilm inhibition Fig. 5a. The nanocomposites, on the other hand, have superior antibacterial activity against *E. coli* and *S. aureus*, as shown in the zone of inhibition photographic images and bar diagram of zone of inhibition values in Figs. 5b and 6 b^1^ and 6 b^2^. Because of the thin layers of gram-negative bacteria and nanocomposite was easily penetrated into cell walls, *E. coli* has the highest antibacterial agents than *S. aureus*. Figs. 5b and 6 b^1^ and 6b^2^show the zone of inhibition values determined for treated ZnO–MnO_2_, Kaol/Chit ZnO–MnO_2_/Kaol/Chit NCs (10, 15, 20 and 30 μg/ml) nanoparticles. Although treatment with ZnO–MnO_2_ and Kaol/Chit nanocomposites alone was ineffective, the ZnO–MnO_2_/Kaol/Chit NCs conjugated with low concentrations of individual NCs had improved antibacterial properties. The results showed that the biocompatibility and dispersion ability of nanocomposites were enhanced by altering their morphological structure. This was observed because the cell shape was uniformly distributed, suggesting an ideal environment for cell growth. In clinical tissue engineering, the NCs exhibit great promise for promoting osteoblast proliferation and development and for facilitating bone production.

### 3.7 Antioxidant activity

#### 3.7.1 DPPH assay

The Diphenyl picryol-hydrazine (DPPH) assay, which uses ascorbic acid as a standard control, was used to determine the antioxidant activity of ZnO/MnO_2_, Kaol/Chit NCs and ZnO/MnO_2_/Kaol/Chit NCs as shown in Fig. 6. The capacity of ZnO/MnO_2_ and Kaol/Chit NCs and ZnO/MnO_2_/Kaol/Chit NCs to scavenge DPPH and neutralise the free radical is assessed for antioxidant activity. The standard DPPH assay helps determine the antioxidant activity of ZnO/MnO_2_ and Kaol/Chit NCs and ZnO/MnO_2_/Kaol/Chit NCs by observing the colour change of stable DPPH free radicals, which results in DPPH easily transferring an electron or hydrogen atom and converting into a purple to yellow diamagnetic molecule. It can also not be affected by side effects such as chelating metal ions or enzyme inhibition. The reduced form of DPPH is generated by composite substances that can represent oxygen atoms, causing the solution to lose its violet colour (discoloration), indicating the composite's scavenging potential.

**Fig. 7:**
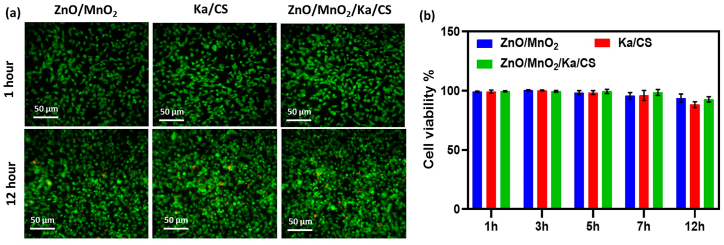
**a:** Up to 12 h of confocal fluorescence microscopy of ZnO/MnO_2_, Ka/CS, and ZnO/MnO_2_/Ka/CS NCs. The colour green indicates that cell growth is viable. **b:** The effect of synthesised ZnO/MnO_2_, Ka/CS, and ZnO/MnO_2_/Ka/CS NCs on NIH 3T3 cell viability. The results revealed a gradual increase in cell viability as well as high biocompatibility for ZnO/MnO_2_, Ka/CS, and ZnO/MnO_2_/Ka/CS NCs. (For interpretation of the references to colour in this figure legend, the reader is referred to the Web version of this article.)

In this study, the absorbance decrease in the DPPH intensity peak at 517 nm was used to assess the composite's free radical scavenging activity. The IC50 parameter, which indicates the ability to change transfer of electrons, has been used to determine the antioxidant properties of ZnO/MnO_2_ and Kaol/Chit NCs and ZnO/MnO_2_/Kaol/Chit NCs synthesised via hydrothermal method. Fig. 7a compares the DPPH free radical scavenging activity (percent) of ZnO/MnO_2_ and Kaol/Chit NCs and ZnO/MnO_2_/Kaol/Chit NCs to ascorbic acid at various concentrations (20, 50, 75 and 100 μg/mL). The results showed that with increasing concentration, the radical scavenging values of the synthesised ZnO/MnO_2_ and Kaol/Chit NCs and ZnO/MnO_2_/Kaol/Chit NCs increased by 42.3 %, 63.2 %, 79.3 %, and 82.4 % [10]. The IC50 inhibition values for control and ZnO/MnO_2_ and Kaol/Chit NCs and ZnO/MnO_2_/Kaol/Chit NCs were 1 μg/mL. The obtained ZnO/MnO_2_/Kaol/Chit NCs displayed significant antioxidant activity.

### 3.8 Evaluation of *in vitro* wound healing

Fig. 7, the biocompatibility of ZnO/MnO_2_, Kaol/Chit and ZnO/MnO_2_/Kaol/Chit NCs was evaluated in terms of *in vitro* cell viability as measured by mitochondrial activity on NIH 3T3 like fibroblasts at various concentrations using the MTT assay. Because it regulates fibrin clot, collagen synthesis, ECM, and wound contraction, the fibroblast is an important cell in wound healing. Several factors influence the cell viability of nanocomposite ZnO/MnO_2_, Kaol/Chit and NCs on biological cells (known as biocompatibility). Mammalian cells were toxic to ZnO/MnO_2_/Kaol/Chit NCs. Particle dissolution and the shedding of intracellular Zn^2+^, Mn^2+^, Al^3+^, Si^4+^ and O^4−^ are the causes. In order to assess cytotoxicity, the cell viability of NIH 3T3 cells treated with ZnO/MnO_2_, Kaol/Chit, and ZnO/MnO_2_/Kaol/Chit NCs was measured using confocal fluorescence microscopy for up to 12 h (Fig. 7b). Approximately 97.58 % of viable cells were observed after 7 h of ZnO/MnO_2_/Kaol/Chit NCs. The effect on cell viability is proportional to the number of days added. The better cell viability and relatively large surface area of the ZnO/MnO_2_/Kaol/Chit NCs were due to the exfoliation of ZnO/MnO_2_ NCs decorated into Kaol/Chit NCs between the free organic functional groups of ZnO/MnO_2_ NCs and the inorganic moieties present in the ZnO/MnO_2_/Kaol/Chit NCs. For wound area samples, cell viability grew faster in 7 h than it did in 12 h in Fig. 7a. Furthermore, due to the large surface area of NCs materials have a highly positive charge and the cell membrane having a predominantly negative charge, NCs materials are more optimised in 7 h. The negatively charged cell membrane was too low and proportional to growth after 7 h.

## 4 Conclusion

In summary, ZnO/MnO_2_/Ka/CS NCs can be cost-effective, eco-friendly, and quickly synthesised using a hydrothermal method. The results of XRD patterns revealed a NCs arrangement with average crystalline sizes of 30 nm, 35 nm and 25 nm, respectively, and established that formation of nanocrystalline particles into the composited form. The morphological analyses of NCs demonstrated that slight decrease in average particle size (20.66 nm) when compared to the shape and size of comparative samples. The increased antibacterial efficacy of NCs coated with ZnO and MnO_2_ NPs against S. aureus and E. coli was attributed to the combined effects of the NCs. This conclusion was supported by the results of the zone of inhibition assay and biofilm assay. The biocompatibility of the NCs was assessed using *in vitro* cytocompatibility experiments employing fluorescent live/dead assays with fibroblast cells. The results of these assays demonstrated that the NCs exhibited a much higher level of biocompatibility compared to the ZnO/MnO_2_ and Ka/CS components. Therefore, the combination of antibacterial properties in the ZnO/MnO2 nanocomposite, together with its enhanced biocompatibility, suggests that this composite has the potential to be advantageous in the development of antibiotic products, specifically for application in wound dressings.

## Funding

None.

## Data availability statement

Data will be made available on request.

## CRediT authorship contribution statement

**Xia Cheng:** Writing – original draft, Supervision, Project administration. **Jingjing Yao:** Investigation, Conceptualization. **Wenhao Fan:** Software, Methodology, Formal analysis. **Li Zhen:** Writing – review & editing, Validation, Resources, Data curation.

## Declaration of competing interest

The authors declare the following financial interests/personal relationships which may be considered as potential competing interests:There are no conflicts of interest for the present research work. If there are other authors, they declare that they have no known competing financial interests or personal relationships that could have appeared to influence the work reported in this paper.
